# Construct validity of a global scale for Workplace Social Capital based on COPSOQ III

**DOI:** 10.1371/journal.pone.0221893

**Published:** 2019-08-29

**Authors:** Hanne Berthelsen, Hugo Westerlund, Jan Hyld Pejtersen, Emina Hadzibajramovic

**Affiliations:** 1 Centre for Work Life and Evaluation Studies (CTA) & Faculty of Odontology, Malmö University, Malmö, Sweden; 2 The Stress Research Institute, Stockholm University, Stockholm, Sweden; 3 VIVE–The Danish Center for Social Science Research, Copenhagen, Denmark; 4 The Institute of Stress Medicine, Region Västra Götaland, Gothenburg, Sweden; 5 Health Metrics, Department of Public Health and Community Medicine, Institute of Medicine, Sahlgrenska Academy, University of Gothenburg, Gothenburg, Sweden; University of Copenhagen, DENMARK

## Abstract

**Background and aim:**

Workplace Social Capital has been suggested as a useful concept when addressing organizational and social factors of the work environment. The overall aim of the present study is to establish and evaluate the construct validity of a measure of Workplace Social Capital based on the operationalization suggested in the third version of the Copenhagen Psychosocial questionnaire.

**Methods:**

The present study is based on data collected as part of a validation and development project for the use of the Swedish version of COPSOQ at workplaces and includes responses from 1316 human service workers answering a workplace survey. Six items from scales for organizational justice, vertical trust and horizontal trust in COPSOQ III were included in the analyses. Rasch Analysis was used for scale validation.

**Results:**

The analyses showed that the psychometric properties of the suggested COPSOQ scale for Workplace Social Capital were satisfactory after accommodation for local dependency. Each individual item worked as intended, the scale was unidimensional and functioned invariantly for women and men, and for younger and older employees. The scale was furthermore found to be valid for use for distinguishing groups, not individuals.

**Conclusion:**

We have established that the scale for Workplace Social Capital measured by COPSOQ III is valid for distinguishing groups, e.g. work teams. The scale exhibits good construct validity as it satisfies the measurement criteria defined by the Rasch model.

## Introduction

In this study we use modern test theory to evaluate the construct validity of a global scale for Workplace Social Capital, based on the operationalization introduced in the third version of the Copenhagen Psychosocial Questionnaire, COPSOQ III [1].

The Nordic labor markets are characterized by a high degree of unionization density and a tradition of employer and union collaboration on work environment issues [2]. Typically, workplaces in the Nordic countries employ participatory approaches and managerial support for a healthy work environment rather than a conflict culture [3] Work-related stress and health complaints remain an internationally unresolved issue [4]. This applies also to the Nordic context, even though stress is problematic from the perspective of both the individual and the organizations. In the National Swedish Work Environment Survey from 2015 half of the employees reported too high a work load and four out of ten found their work situation mentally distressing; these problems were most pronounced among human service workers [5]. In 2016, new provisions were introduced in Sweden in order to improve the handling of occupational safety and health at workplaces by means of a more holistic and comprehensive approach addressing the organizational and social work environment [6]. Another initiative was the establishment of the governmental “Trust Delegation” [7] with an overall aim of promoting trust-based organizational development in municipalities and projects, including the total governance process. This Swedish re-orientation is in line with the growing attention paid to addressing upstream factors of work life and including perspectives from positive occupational psychology in the Nordic countries in general [8]. However, according to Saksvik et al. there is a strong need for organizational-level social relations to be defined and measured as workplace norms [9]. In sum, this makes social capital an important concept to measure as a workplace characteristic rather than an individual characteristic.

Social capital was introduced in the social sciences in the mid-eighties, (see e.g. [10–12]). In his seminal work “Bowling alone” [13], Putnam reflects on American society and notes an erosion of social capital, understood as “features of social organization such as networks, norms, and social trust that facilitate coordination and cooperation for mutual benefit”. Putnam describes trends of declining social connectedness, civic engagement and civic trust in social life and wonders whether there is an equilibrium of social capital where it develops in other domains of everyday life, such as at workplaces. A decade later, at the beginning of the new millennium, Nordic work life researchers picked up the thread and introduced social capital as a useful concept in a workplace context (see for example [14–18]). Questionnaire items covering Workplace Social Capital were developed and in 2004–05 they were integrated in the Danish national work environment survey that led to establishment of the second version of the Copenhagen Psychosocial Questionnaire, COPSOQ II [19]. The items regarding Workplace Social Capital covered aspects of trust and justice and were inspired by researchers in the field, including colleagues from Finland [19–22]. The latest Danish Psychosocial Questionnaire has likewise included social capital [23]

### Operationalization of Workplace Social Capital by the COPSOQ instrument

Workplace Social Capital has been operationalized based on COPSOQ II in a number of studies. In order to gain an overview we conducted a literature search, and identified 13 articles–published in international peer-reviewed journals–which have all operationalized social capital by COPSOQ II for analytical purposes. The search was conducted on June 1, 2018, using the string: “COPSOQ AND “social capital”” in Google Scholar. The search was replicated in PubMed and Scopus without yielding additional hits.

The overview presented in Table 1 reveals that the core of the measurement across studies is based on combinations of trust and justice items. The items belonging to the trust and justice scales were specifically designed to catch a broader perspective than the individuals’ own perspective [19]. The validity of measurement of Workplace Social Capital as a group construct was further corroborated by cognitive interview results [24]. One research group has chosen to replace the entire justice dimension with a scale for Rewards, which basically asks about the respondents’ individual perception of being recognized, respected and treated fairly at work rather than addressing work climate [9]. In addition to trust and justice, the scale for Community at work is frequently included, in particular the item SW2 addressing whether there is good cooperation between colleagues at work (Table 1). Additionally, three of the research groups have chosen to further include items from other scales or instruments. The majority of the articles (10 out of 13) were published during 2016–18, which can be seen as a further indication of a growing interest in including the construct in work life research. The third version of the COPSOQ instrument presents a domain for Workplace Social Capital, which includes the scales for Vertical Trust, Horizontal Trust and Organizational Justice [1]. The domain is in accordance with the core of previous operationalizations as presented here.

**Table 1:** Overview of internationally published studies where Workplace Social Capital has been operationalized by COPSOQ II for analytical purposes.

Reference	COPSOQ II items					Non-COPSOQ items
	Trust	Justice and respect	Community at work	Rewards	Other	
[25]	TE3	JU1, JU2, JU3, JU4	SW1, SW2, SW3			4 items on organizational climate from the Finnish ‘Healthy Organizations Barometer’
[26]	5 reformulated items	2 items			1 item on influence	3 ad hoc items on cooperation
[27]	6 unspecified items from the three scales on trust and justice					
[28–30]	TM2, TM3 TE2, TE3			RE1, RE2, RE3		From the Modern Work Life Questionnaire
[24]	TM1, TM2, TM3, TM4, TE1, TE2, TE3	JU1, JU2, JU3, JU4				
[31]	TM1, TM2, TE3	JU1, JU4	SW2	RE1		3 items on cooperation in accordance with the note from Pejtersen et al. 2011.
[32]	TM2, TM4, TE3	JU1, JU2, JU4	SW2			
[33]	TE2, TE1,	JU1, JU4		RE1		
[34]*	1 item	1 item				
[35]	TM1, TM2	JU1, JU4				4 items on social support, respect, and co-worker responsibility
[36]	TM1, TM2,	JU1, JU4				4 items on social support, respect, and co-worker responsibility

Abbreviations: Vertical Trust (TM), Horizontal Trust (TE), Organizational Justice (JU), where M denotes Management and E Employees. The variable names follow those presented in the COPSOQ II position paper by Pejtersen et al. [19].

* Workplace Social Capital operationalized as an independent variable used for the purpose of evaluating convergent validity of the Work-Wellbeing index. One item on trust and one on justice (both items reformulated, not possible to identify the items based on the appendix of the publication).

Workplace Social Capital is a latent construct meaning that it is not directly observable and therefore cannot be measured directly. A latent construct is measured through indicators (items) that represent the underlying construct. Consequently, it is more complicated to measure in a reliable and valid way than directly observable variables. Operationalization of latent constructs relies on theories and multi-item questionnaires are often used to capture the underlying construct which is considered to be the cause of the item responses.

Before it was launched, the COPSOQ II instrument was validated in a number of ways and presented in a theme issue of Scandinavian Journal of Public Health, 2010. In addition to classical test theory methods, modern test theory such as differential item functioning was also used, although not for the social capital scales [37]. In a note from 2011 Pejtersen, Björner and Hasle from the National Research Centre for the Working Environment, Denmark, reported results on psychometrics of measures of Workplace Social Capital based on the scales for Vertical Trust, Horizontal Trust, Justice and Respect and items regarding Organizational Citizenship Behaviour [38]. However, this work was not further developed for international publication. Consequently, the only validation study published on Workplace Social Capital operationalized by the COPSOQ instrument so far is the qualitative study corroborating the content validity of the items on trust and justice [24].

Usually, in multi-item questionnaires each item has a set of several mutually exclusive response options that can be classified as ordinal variables. Ordinal variables are numerically coded showing frequency, magnitude etc. so that responses can be ordered from lowest to highest. However, the distances between the response categories are unknown, thus these values do not have the mathematical properties needed for arithmetic calculations. Interval scales, on the other hand, ascribe hierarchy and denote numerical differences that reflect the differences between the objects. The intervals between each value on an interval scale are equal.

Once a questionnaire is constructed, the soundness of data the collected by means of questionnaires is judged by the measurement properties, i.e. validity and reliability. Validity refers to the ability of an instrument to measure what it is intended to measure, while reliability can be understood as accuracy or the extent to which repeated measurements lead to similar results.

A standard procedure for handling data from questionnaires is to construct a global score based on the item responses to represent a latent construct. Unidimensionality is a prerequisite for combining the items into a single global score. Calculating the mean values of the item responses is a very common procedure. However, due to the non-metric properties of ordinal data, this procedure should not be taken for granted. An important aspect of validation is the adequacy of the scaling of scores which can be done by modern psychometric techniques such as Rasch Analysis [39].

The aim of the present paper is to use Rasch Analysis to examine the internal construct validity of a global scale for Workplace Social Capital based on COPSOQ III items.

## Material and methods

### Study design and population

The present paper is based on cross-sectional data collected during the period from November 2016 to October 2017 as part of a validation and development project for the use of the COPSOQ instrument at workplaces. Workplaces were offered a free anonymous work environment survey through the Swedish COPSOQ webpage, based at Malmö University. All staff members at the workplaces received a link to an online questionnaire, in an email with an introduction from the workplace itself and information about the research project. Each survey was open 3–4 weeks and included two reminders. The overall response rate for the convenient sample of workplaces included in the present study was 83.4% (ranging from 63.5% to 95.0%).

For the purpose of the present study we considered data from 1426 employees from 10 public and private human service organizations. Inclusion criteria were non-managerial employees stating that they have direct contact with patients, clients, pupils etc. as part of their job and with a job title indicating human service work (e.g. social workers, dentists, physicians, nurses, psychologists, physiotherapists, teachers). Only employees with complete data on all variables included in the present study were considered for the analyses, which resulted in 1316 employees being available (211 men and 1105 women).

For evaluation of differential item functioning (DIF) in a Rasch Analysis (explained under data analyses), the groups compared should be of approximately equal size [40]. The rationale behind this recommendation is to ensure that if there is DIF, one group does not dominate in the estimates of parameters. Consequently, to achieve a balanced data set in terms of age, age was dichotomized into two groups: up to 44 years and 45 years or more. To obtain a balanced data set with respect to gender, all 211 men were included and 211 women were randomly sampled out of a total 1105 women with complete data.

Thus, in the present study 422 individuals were included. This sample size is large enough to give a high degree of precision, i.e. item location estimates within 0.3 logits with 99% confidence for the Rasch Analysis [41].

For the sample, 54% (229) of the employees were up to 44 years old and 46% (193) were 45 or older, and the vast majority of employees were professionals (based on the International Standard Classification of Occupations (ISCO) group 2).

### Measures

Workplace Social Capital was measured by items from the scales for *Vertical Trust*, *Horizontal Trust* and *Organizational Justice* based on the international middle long version of the COPSOQ III (Table 2) [1]. All six items had the following response options: To a very small extent, To a small extent, Somewhat, To a large extent, To a very large extent. For analytical purposes each item was coded 0–25–50–75–100, following the standard COPSOQ scoring [1].

**Table 2:** Overview of items (variable name refers to international COPSOQ III nomenclature [1]).

Item	Wording in English	Wording in Swedish
TM1	Does the management trust the employees to do their work well?	Litar ledningen på att medarbetarna gör ett bra jobb?
TMX2	Can the employees trust the information that comes from the management?	Kan medarbetarna lita på den information som kommer från ledningen?
TM4	Are the employees able to express their views and feelings?	Är det möjligt för medarbetarna att uttrycka sina åsikter och känslor?
TE3	Do the employees in general trust each other?	Litar medarbetarna i allmänhet på varandra?
JU1	Are conflicts resolved in a fair way?	Löses konflikter på ett rättvist sätt?
JU4	Is the work distributed fairly?	Fördelas arbetsuppgifterna på ett rättvist sätt?

The items are based on three sub-scales, of which two deal with trust in work relations and one with organizational justice. Our a priori expectation was that this could be reflected in the dimensionality and in the mutual hierarchy of items. According to Holtz [42] and Colquitt and Rodell [43], the tradition in management research has been to regard trust as an outcome of justice, drawing on, for example, social exchange theory [44] or fairness heuristics theory [45]. However, such a simple causal order can be questioned, as argued by Shapiro and Kirkman, who introduced the principle of anticipatory justice [46]. Recent organizational and psychological experimental studies corroborate that trust can shape the expectations of justice [47, 48]. Based on this line of research our a priori expectation was that the item on mutual trust among employees (TE3) would be endorsed most easily, followed by the items regarding vertical trust (TM) and that the organizational justice items (JU) would be the highest ordered in the mutual hierarchy. This follows a reasoning that people can have a sense of whether relationships are characterized by trust independently of concrete experiences, while the COPSOQ III items regarding organizational justice ask more directly about the handling of potentially problematic situations.

### The Rasch model and data analysis

We tested whether the data fitted the Rasch measuring model [39]. The model is applied in the development and evaluation of measurement properties of multi-item questionnaires. The purpose is to provide a global score that is a sufficient statistic for the latent construct that is being measured by the questionnaire. According to Rasch, latent constructs measured by the questionnaire (e.g. Workplace Social Capital) should have properties analogous with physical measurements, with positive real numbers defined as regularly as the measurement of height, and not through some arbitrary grading scale. In this way fundamental or objective measurement can be achieved. An important property of fundamental measurement is that it allows for arithmetic operations such as addition and subtraction. The Rasch model operationalizes the axioms of additive conjoint measurements, which are the requirements for the fundamental measurement construction [49–52]. The process of a Rasch Analysis is concerned with whether or not the data meets the model expectations. The adequacy of the fit is evaluated by means of multiple tests of summary fit statistics (overall fit to the model), individual item and person statistics, as well as graphical examinations of fit.

In the present study, the data were fitted to the Rasch measurement model using the unrestricted or partial credit model for polytomous cases, which allows the distances between thresholds to vary across the items [53, 54]. A threshold is the point between any two adjacent categories in which the probability of either response is equally likely. Data were fitted to the Rasch model using the RUMM2030 software [55]. Assumptions of unidimensionality, monotonicity, local dependence and differential item functioning (DIF) were tested, briefly explained below [56].

The overall fit to the model was evaluated by the item-trait interaction (χ^2^ statistic), and mean person and item fit residuals. The item-trait interaction statistic tests the property of invariance across the latent trait and a significant value indicates that the hierarchical ordering of the items varies across the latent continuum. The mean person and item fit residuals are expected to be close to zero with a standard deviation (SD) of one. A non-significant value of the χ^2^ statistic reflects the property of invariance across the trait. The invariance criterion implies that the items need to work in the same way (invariantly) across the whole continuum of the latent construct for all individuals. Given the same level of the latent trait (e.g. Workplace Social Capital), the scale should also function in the same way for all comparable groups (e.g. gender or age). This is commonly known as no differential item functioning (DIF). Monotonicity implies that the item responses are positively related to the latent variable. The response structure required by the Rasch model is a stochastically consistent item order, i.e. a probabilistic Guttman pattern [57]. This means that persons who experience higher levels of Workplace Social Capital are expected to get higher scores, whereas persons with lower levels of Workplace Social Capital are expected to get lower scores. The intended increasing level of Workplace Social Capital across the response categories for each item needs to be reflected in the observed data. The reliability of the scale is reported as a Person Separation Index (PSI), i.e. the proportion of true variance to true and error variance. Values of 0.7 and 0.9 are indicative of sufficient reliability for group and individual use respectively [58].

Local dependency (response and trait dependency) was investigated by residual correlations [59]. The trait dependency violated the assumption of unidimensionality, which was tested by Smith’s test of unidimensionality [60]. For this test, first principal component analysis (PCA) on residuals was performed. Then items loading positively and negatively on the first principal component of the residuals are used to make independent person estimates. Next, independent t-tests for the differences in these estimates for each persons were performed [60]. Less than 5% of such tests outside the range of –1.96 to 1.96 supports the unidimensionality of the scale. A 95% binomial confidence interval of proportions [61] was used to show that the lower limit of the observed proportion was below the 5% level [60]. Possible local dependency can be accounted for by combining correlated items into testlets where the items are added together and then comparing the model fit with that provided by the initial analysis [62].

Besides overall fit, individual item fit was evaluated by item’s χ^2^ statistic, the item’s ability to discriminate (item fit residuals expected to be within the range ±2.5), the appropriateness of the response categories (threshold ordering), response dependency relative to other items (residual correlations 0.2 above the average correlation) [63]) and the absence of DIF for gender and age. DIF was tested by conducting ANOVA of standardized residuals. The disordering of the thresholds was examined graphically by plotting category probability curves. Disordering of the thresholds was tested by the hybrid approach proposed by Salzberger [64].

Evaluation of the targeting, i.e. the distribution on a logit scale, of the items and persons in the sample was examined graphically by a person item threshold distribution graph. For a well-targeted instrument, the mean location for persons would be around the value of zero. In the event of good fit, Rasch person estimates, which are logits, can be transformed into a convenient range (henceforth referred to as metric score) [65].

Data is available from Appendices 1–2.

## Results

The frequency distribution for each item is presented in Table 3. For all items, the majority of the responses were concentrated in the categories *Somewhat* and *To a large extent*, while the lowest response category (*To a very small extent*) was rarely used.

**Table 3:** Frequency distribution of Workplace Social Capital items of the COPSOQ III.

	To a very small extent	To a small extent	Somewhat	To a large extent	To a very large extent
**Item**	**% (n)**	**% (n)**	**% (n)**	**% (n)**	**% (n)**
TM1	1 (5)	4 (18)	20 (82)	50 (203)	25 (102)
TM2	2 (7)	5 (22)	28 (116)	48 (196)	17 (69)
TM4	4 (16)	6 (24)	25 (101)	50 (204)	16 (65)
JU1	4 (17)	7 (29)	40 (163)	41 (170)	8 (31)
JU4	2 (9)	8 (34)	35 (142)	49 (200)	6 (25)
TE3	<1 (1)	3 (11)	20 (81)	56 (231)	21 (86)

Initially, the Rasch Analysis was performed on all six Workplace Social Capital items. The summary fit statistics of the initial analysis are shown in Table 4, analysis 1. The fit to the model was good according to non-significant χ^2^ statistic. The PSI value was 0.83, which is above the predefined criterion of 0.7 valid for group comparisons. Item and person fit residual values were somewhat higher than the ideal values shown at the bottom of the table.

**Table 4:** Fit to the Rasch model, summary fit statistics, n = 422.

		Item residual		Person residual		Chi square		Unidimensionality	
Analysis name		Mean	SD	Mean	SD	Value	p	PSI	Test % (95% CI)
1	SocCap, 6 items	–0.63	1.94	–0.52	1.17	46.93	0.11	0.83	8.8 (6.4;11.9)
2	SocCap, 2 testlets	–0.08	0.96	–0.48	0.78	16.09	0.19	0.78	3.2 (1.8;5.4)
	Ideal values	0.0	<1.4	0.0	<1.4		>0.05	>0.7	(LCI <5%)

Acronyms. SD = Standard deviation, PSI = Person Separation Index, SocCap = Social Capital scales of the COPSOQ III.

With respect to individual item fit, all items besides TM4 had item fit residual values within the predefined optimal range of ±2.5 and a non-significant chi-square statistic (Table 5). The fit residual value for item TM4 was –3.23, although this was not statistically significant (χ^2^ = 7.69, p = 0.26). In addition, item locations in hierarchical order are shown in Table 5.

**Table 5:** Item locations, standard errors, item fit residuals, item Chi Square statistics, degree of freedom and p-values for the six items of the Workplace Social Capital scale of the COPSOQ III.

Item	Location	SE	FitResid	ChiSq/DF	p-value
TE3	–0.78	0.083	1.51	7.05/6	0.32
TM1	–0.54	0.076	–2.04	6.60/6	0.36
TM2	–0.10	0.076	–1.72	8.42/6	0.21
TM4	0.25	0.07	–3.23	7.69/6	0.26
JU4	0.47	0.077	0.81	11.03/6	0.09
JU1	0.71	0.072	0.87	6.13/6	0.41

Acronyms. Standard errors (SE), item fit residuals (FitResid), item Chi Square statistics (ChiSq), degree of freedom (DF)

No items showed DIF for gender or age. High residual correlations were not observed for any pair of the items.

**Fig 1:**
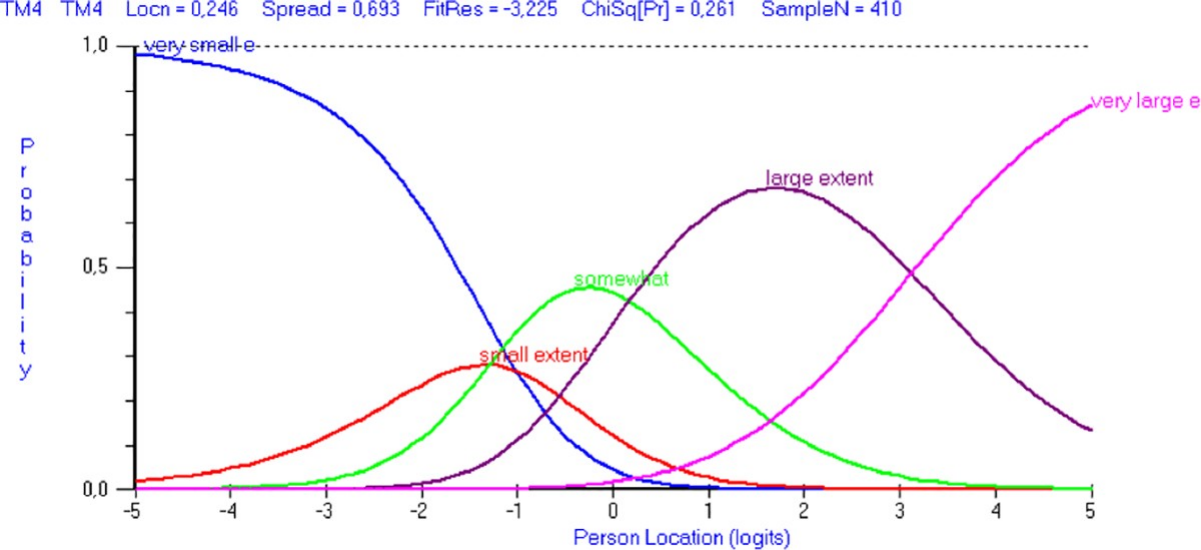
Category probability curves. Category probability curves for item TM4 with response options: To a very small extent, to a small extent, somewhat, to a large extent, to a very large extent.

Two items (TM4 and JU1) had disordered thresholds, i.e. indicating that it may be difficult for respondents to distinguish between response categories. As seen in Fig 1, showing category probability curves for item TM4, disordering was observed for the first two thresholds. The same pattern was observed for JU1. Disordering of the thresholds was not significant according to Salzberger’s hybrid approach. To rule out the possibility of disordering due to the few observations in the lowest response categories, an additional sensitivity analysis was performed by rerunning the analysis on the original sample of 1316 participants. In that analysis, TM4 and JU1 had ordered thresholds (results not shown).

The PCA conducted on item residuals revealed that the three TM items loaded positively on the first principal component, while the reverse was true for the items TE3, JU1 and JU4. These two groups of items were then used to perform Smith’s test of unidimensionality, which revealed problems with local dependency as the lower CI was 6.4% (Table 4, analysis 1). An attempt to resolve this issue was done by performing an additional analysis by combining the positively and negatively loaded items into two testlets (Table 4, analysis 2). As seen by the summary fit statistics, fit to the Rasch model was achieved. Accounting for local dependency by testlets resulted in a decreased PSI value (0.78 compared to 0.83). Additional sensitivity analysis was done by performing the Rasch Analysis on the entire sample (n = 1316) and the results were similar. The summary fit table for this analysis is found in Appendix 3.

**Fig 2:**
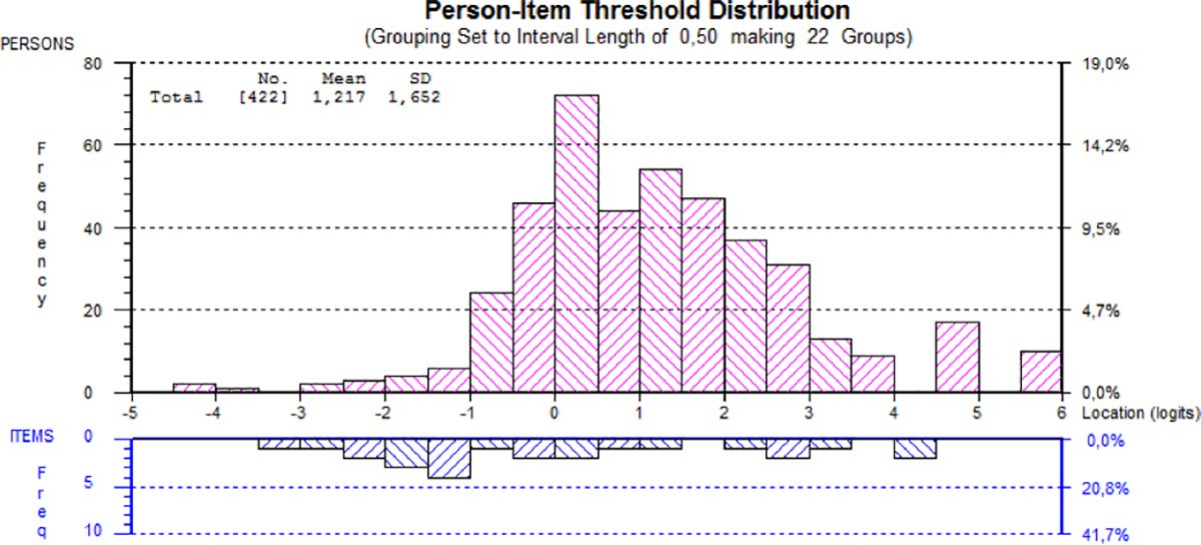
Person and item threshold distribution along the logit scale, analysis on two testlets. Higher values indicate higher Workplace Social Capital within the Workplace Social Capital scale of the COPSOQ III.

The distribution of the items and persons along the common logit scale (higher values indicate higher Workplace Social Capital) is shown in Fig 2, indicating satisfactory targeting, although the mean of the persons (1.2) was higher than the mean of the items (constrained to zero).

### Ordinal-to-interval conversion table

Given the fit to the Rasch model, ordinal scores (mean values of the six items) are transformed into interval-level scores. Interval-level scores from the Rasch model are on the logit scale, which can take both negative and positive values, with higher scores indicating higher levels of Workplace Social Capital. These logits scores are then transformed into a 0–100 interval, which is a more familiar range for COPSOQ users (henceforth referred to as a metric score). Table 6 provides interval scores in both logit unit and in a 0–100 range that allows users of the Workplace Social Capital scale to convert ordinal mean score into interval-level (metric) scores ranging 0–100.

**Table 6:** Conversion table. Conversion table with interval scale in logit and metric (linearly transformed logit into 0–100 range) on the COPSOQ III Workplace Social Capital scale and their corresponding ordinal scale (mean) equivalents based on Rasch analysis with two testlets, n = 1316.

Logit (SE)	Metric	Mean	Logit (SE)	Metric	Mean
–3.0 (0.90)	0	0	–0.2 (0.52)	36.1	54.2
–2.5 (0.65)	5.9	4.2	0.1 (0.54)	39.9	58.3
–2.2 (0.53)	10	8.3	0.4 (0.56)	44.0	62.5
–2.0 (0.47)	12.8	12.5	0.7 (0.57)	48.2	66.7
–1.8 (0.43)	15.0	16.7	1.1(0.58)	52.5	70.8
–1.7 (0.41)	17.0	20.8	1.4 (0.59)	57.0	75.0
–1.5 (0.40)	18.7	25.0	1.7 (0.61)	61.5	79.2
–1.4 (0.40)	20.5	29.2	2.1 (0.64)	66.3	83.3
–1.3 (0.40)	22.3	33.3	2.5 (0.69)	71.6	87.5
–1.1 (0.42)	24.2	37.5	3.0 (0.78)	78.3	91.7
–0.9 (0.44)	26.5	41.7	3.7 (0.97)	87.4	95.8
–0.7 (0.47)	29.2	45.8	4.7 (1.33)	100	100
–0.5 (0.50)	32.5	50.0			

Researchers and practitioners who have already used the Workplace Social Capital scale of COPSOQ III to collect data or are planning to use the scale can apply the results of this study as follows. First calculate the raw mean score of the six items coded 0–25–50–75–100 (based on complete datasets). Next, use Table 6 to convert these scores into the corresponding metric scores.

**Fig 3:**
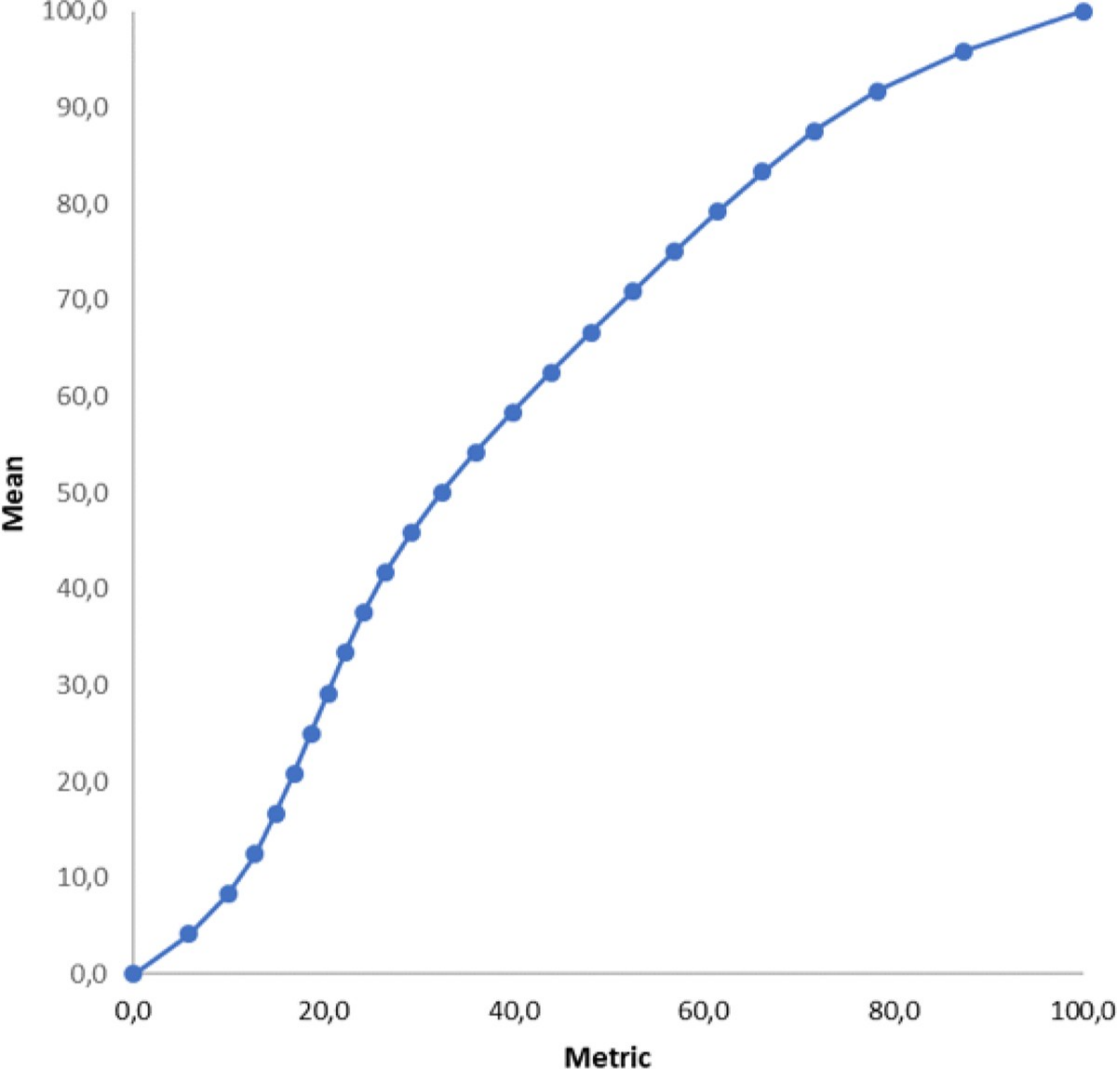
Comparison of the metric and mean score for the Workplace Social Capital scale of the COPSOQ III.

An interval-level scale is equidistant, meaning that the increase of e.g. 5 points on a scale implies the same magnitude of increase in Workplace Social Capital on the entire range of the scale. As seen in Fig 3, where mean scores (Y-axis) are plotted against metric score (X-axis), this assumption does not hold for the ordinal-level scale. Clearly, the curve is not a straight line. The increase of e.g. one unit on a mean scale is much larger at the top than in the middle of the scale. Using the conversion table (Table 6) allows for increased precision of the Workplace Social Capital scale.

## Discussion

We found that the psychometric properties of the suggested scale for Workplace Social Capital were satisfactory after having accommodated for local dependency. Each individual item worked as intended, the scale was unidimensional and functions invariantly for women and men, and for younger and older employees. The scale for Workplace Social Capital is valid for use at group level, understood as valid for distinguishing groups, not individuals.

The psychometric properties of the Workplace Social Capital scale from COPSOQ III were good, having accommodated for local dependency, through the use of testlets and all items worked properly. The ordering of the response categories worked properly for all six items, meaning that the expected increasing level of the latent construct (i.e. Workplace Social Capital), across the response categories for each item, are reflected in the observed data. The results suggest that the scale fulfills the criteria required by the Rasch model in order to quantify a latent variable. The total score reflects the scoring structure indicated by developers of the scale. In other words, the Rasch model provides evidence that the items form a reasonably unidimensional and essentially valid scale. An essential feature of measurement implies equal intervals across the entire continuum of the construct being measured.

The initial analysis showed some problems with local dependency. Based on the PCA of residuals, the three TM items clustered in one group while the two JU items and TE3 clustered in another group. Although this is not optimal from a measurement point of view, the results are not surprising from a theoretical point of view, since these items constitute different subdimensions of social capital. While the three TM items are intended to measure the perception of trust in the relationship between management and employees, the TE3 and JU1, JU4 items address mutual trust among employees and justice as group-level characteristics, as reflected in quotations from qualitative interviews [24]. This might be especially true in the context of human services, where employees typically have high discretion in tasks related to clients or patients, including handling of conflicts and distribution of tasks [66]. The measurement problem was solved by combining the items into two testlets, which is a recommended procedure for this kind of issue [62].

Equal spacing between the scale categories is an underlying assumption for the mean scores to be meaningful. We have shown that the mean scores of Workplace Social Capital scale are not linear and do not have equal intervals along the whole continuum. An increase of one unit on a mean score scale does not imply the same amount or magnitude of Workplace Social Capital across the entire continuum. The problem might not be that serious in the middle of the scale but is more pronounced towards the ends of the scale. This is not in any way unique for the Workplace Social Capital scale but is true for many ordinal scales. Although it is tempting and very common to use the numerical coding of ordinal variables as real numbers in statistical analysis, the numerals assigned to the response categories are arbitrary and can be changed as long as their ordering is preserved [67, 68]. A discussion about how to treat ordinal data in statistical analysis has been going on for a long time [69] and classical test theory and modern test theory offer different solutions. However, that discussion is beyond the scope of this article.

The ordinal mean score has been transformed to an interval scale latent estimate. We recommend the use of the proposed metric score instead of the mean score. The logit score is transformed to the 0–100 interval (called metric score), which is a familiar range for COPSOQ users and where higher scores indicate higher Workplace Social Capital. A task for future studies will be to determine cut-off scores indicating high and low values of Workplace Social Capital as well as relevant reference values for workplaces.

The results of this study showed that the scale is valid on a group level but not for individual use, e.g. to follow an individual over time. This is also in line with the theoretical basis of Workplace Social Capital, which deals with relationships at work ([16, 17, 19, 70]). In a review of the literature and guidance on measurement of Social Capital in general, Lochner, Kawachi and Kennedy point out that ‘*an almost universal agreement is that community characteristics* [such as social capital] *ought to be distinguished from individual characteristics*, *and measured at the community level’* [71]. Measurement at the workplace level can be done in different ways, for example by observational studies, document analyses or by questionnaire studies. In accordance with the recommendation from Lochner and colleagues [71], the COPSOQ measurement of Workplace Social Capital can be seen as an ecologic characteristic of the group to which individual responses are aggregated rather than an attribute of the individual [24].

As part of the present work we realized that Workplace Social Capital has been defined and operationalized by the use of COPSOQ items and in numerous ways across different research projects (Table 1). However, in the vast majority of the studies presented in Table 1 we find only very sparse information on validity and reliability, often only Cronbach’s alpha values or a reference to the fact that COPSOQ in general or specific scales are well validated. A basis for all research is the use of reliable and valid instruments, and it is therefore our hope that this article can be a step towards the establishment of consensus regarding measurement of COPSOQ III Workplace Social Capital. Future studies will contribute to further validation of the instrument.

A main advantage of our study is that Rasch Analysis has been used, which belongs to the item response theory and does not require any assumptions about the distribution of the construct being measured. The Rasch model operationalizes the axioms of additive conjoint measurements, which are the requirements for the fundamental measurement construction [49–52]. The adequacy of the fit was evaluated by means of residuals, i.e. the observed item responses were compared to the expected ones. This is an appropriate method given the ordinal character of questionnaire data. Given that the data fit the Rasch model, a valid measurement is obtained and the sufficient statistic for the Workplace Social Capital items is obtained on a logit scale and transformed into the 0–100 range for reasons of convenience. Some limitations should be mentioned. The proposed logit (and metric) scores are valid only for similar populations of human service workers or similar occupations having daily contact with patients, clients etc. The Workplace Social Capital scale fit the model after the items were combined into two testlets. This implies that the proposed metric values are valid provided that there are no missing values on any of the items.

Further research is needed to address construct validity in relation to other important constructs, and for a better understanding of the construct when used in different contexts. According to modern test theory, validation is an ongoing process, and modified versions of the questionnaire at hand, or applied in new settings, call for new evaluations [72]. In the present study we have used a convenience sample of human service workers. Our findings showed that the scale worked satisfactorily and showed an ability to distinguish between groups even in a high-trust context, such as human services. While human service work shares many characteristics across countries, the core of the work differs substantially from, say, industrial work [66]. Therefore, we suggest replication of the study in a broader international sample to assess higher generalizability.

## Conclusions

In conclusion, we have established a scale based on the domain for Workplace Social Capital in COPSOQ III for use at group level in human service organizations. The scale holds good construct validity as it satisfies the measurement criteria defined by the Rasch model.

## Supporting information

S1 File Appendix 1. Scientific data related to the analyses, full sample of 1316 participants.
(XLSX)

S2 File Appendix 2. Scientific data related to the analyses, selected sample of 422 participants.
(XLSX)

S3 File Appendix 3. Fit to the Rasch model, summary fit statistics for the full sample, n = 1316.
(PDF)
