# Supplementary material for: Construct validity of a global scale for Workplace Social Capital based on COPSOQ III
Source: PLoS One. 2019 Aug 29;14(8):e0221893. doi: 10.1371/journal.pone.0221893 (PMC6715184; doi:10.1371/journal.pone.0221893)
Supplement: S3 File — Fit to the Rasch model, summary fit statistics for the full sample, n = 1316. (PDF) [file pone.0221893.s003.pdf]

Appendix 3. Fit to the Rasch model, summary fit statistics, n=1316.

		Item residual		Person residual		Chi square		Unidimensionality	
Analysis name		Mean	SD	Mean	SD	Value	p	PSI	Test % (95% CI)
1	SocCap, 6 items	-0.77	3.04	-0.52	1.20	141.5	<0.0001	0.81	6.0 (4.9;7.5)
2	SocCap, 2 testlets	-0.02	1.30	-0.52	0.79	32	0.02	0.76	4.7 (3.7;6.0)
	Ideal values	0.0	<1.4	0.0	<1.4		>0.05	>0.7	(LCI <5%)

Abbreviations. SD=Standard deviation, PSI= Person Separation Index, SocCap= Workplace Social Capital scale of the COPSOQ III.
